# Passive acoustic surveys for predicting species’ distributions: Optimising detection probability

**DOI:** 10.1371/journal.pone.0199396

**Published:** 2018-07-18

**Authors:** Stiele V. Hagens, Anthony R. Rendall, Desley A. Whisson

**Affiliations:** School of Life and Environmental Sciences, Centre for Integrative Ecology, Deakin University, Geelong, Australia; University of Sydney, AUSTRALIA

## Abstract

Surveying terrestrial species across diverse habitats is important for predicting species’ distributions and implementing conservation actions. For vocalising species, passive acoustic monitoring (PAM) is increasing in popularity; however, survey design rarely considers the factors influencing the timing and occurrence of vocalisations and in turn, how they may influence detectability of the species. Here, we use the koala (*Phascolarctos cinereus*) as a case study to show how PAM can be used to first examine the factors influencing vocalisations, and then use occupancy modelling to make recommendations on survey design for the species. We used automated recording units to monitor koala vocalisations at ten sites between August 2016 and January 2017. The timing of male koala vocalisations was linked to time of sunset with vocalisations increasing two hours prior to sunset and peaking at four hours after sunset. Vocalisations had a seasonal trend, increasing from the early to middle stage of the breeding season. Koala population density and stage of the breeding season had more influence on detection probability than daily sampling schedule. Where population density was low, and during the early stage of the breeding season, 7 survey nights (recording for 6 hours from 20:00h to 02:00h; i.e. the period of peak bellowing activity) were required to be 95% confident of a site-specific absence. Our study provides an approach for designing effective passive acoustic surveys for terrestrial species.

## Introduction

The ability to detect and monitor terrestrial wildlife species is critical for predicting distributions and for managing populations [1, 2, 3]. However, most traditional field survey methods are labour intensive and imperfect detection of species remains a challenge, especially for those species that are cryptic, or that occur at few sites or at low densities [4]. Thus, the development of reliable, efficient and cost-effective monitoring techniques is important [5, 6].

Passive Acoustic Monitoring (PAM) is an emerging technique for monitoring terrestrial species across a diversity of taxa [7]. Autonomous Recording Units (ARUs) that are programmable, small, easily deployed and which can be left in the field for extended periods allow researchers to detect and study vocalising species [2, 8]. Advantages of using PAM include: (i) improved detection probability of species that are rare, visually cryptic, small or nocturnal [2, 9]; (ii) less invasive for animals that are sensitive to physical trapping or other direct survey techniques [9]; (iii) logistically easier where vegetation or terrain is difficult to navigate, or where temporal and spatial scales are large [7]; (iv) reduced time spent in the field because ARUs only require installation and maintenance over the given study period [10, 11]; and (v) provide a permanent record of the acoustic environment [10]. Furthermore, detection of vocalisations of focal species in recordings can be automated [8], saving time in data processing [10, 12] while providing an objective measure of detection that is unbiased by human observation [13].

In designing passive acoustic surveys, primary considerations are the type of recording unit used, the number of units and deployment configuration; and recording schedule [7, 14]. The acoustic characteristics of the target animal’s vocalisation are a major determinant of the type of microphone and sensor used because recorders are sensitive to a limited range of frequencies [7]. Deployment configuration of recorders relies on knowledge of the autonomous recorder capabilities, acoustic characteristics of the target species, and site characteristics [7, 14, 15]. Before any study, tests should be conducted to determine the detection space of the recorder and thus the area being surveyed [14, 15]. The recording parameters and schedule relies on knowledge of the vocalising behaviour of the target animal and how it varies seasonally or with environmental conditions [14]. When there is only limited understanding of this, ongoing, continuous recording may be undertaken; however, this assumes that ARUs can be regularly maintained, a reliable power supply is available, and that resources are available for the storage and analysis of the large volume of recordings generated. Consequently, many studies subsample the soundscape using a systematic schedule during the season when the species is known to vocalise. For example, in a study of a threatened warbler, ARUs were programmed to record for one minute per ten minutes for ten days during the breeding period when the species is known to vocalise [16]. However, the probability of detecting the species with a subsampling approach seldom is assessed, and results may lead to errors in predicted distributions.

Here, we demonstrate how the use of PAM for detecting a terrestrial species can be optimised by first obtaining information on how vocalisations vary with population density and seasonal activity of a species, and then using occupancy modelling to determine the optimal sampling schedule relative to these factors. Our study species was the koala *Phascolarctos cinereus*, a vocalising, cryptic, arboreal species that often occurs at low densities (<1 koala per hectare), and which is of conservation concern throughout much of its range [3]. The development of standard protocols for monitoring populations has been identified as a priority for conservation of the species [17]. PAM already has shown promise as a survey method for koalas [18] and may provide an effective, standardised method of monitoring the species, that overcomes the issues of reliability and logistical constraints associated with current survey methods [19, 20, 21].

Vocalisations are most frequent during the breeding season and play an important role in koala social organisation [22], and mate selection [23, 24]. Males produce distinctive ‘bellows’ which have been described as a rasping inhalation followed by a guttural belching exhalation [25, 26]. An additional pair of laryngeal vocal folds allows the pitch of a male’s bellow to be lowered to an average 27.1 Hz, resulting in the bellow broadcasting farther than would be expected for an animal of its size (up to 150m [27]). Female koalas also vocalise, producing ‘screams’, ‘snarls’ or ‘wails’ when approached by males [25, 28]. Such vocalisations make the species an ideal candidate for PAM.

The aim of this study was to develop an approach to designing effective passive acoustic surveys for terrestrial species. Using the koala as a case study, we (i) described the diel cycle and weekly trend in male bellows during the breeding season; (ii) assessed the influence of koala population density, stage of the breeding season (early-, mid- and late-), and a range of sampling schedules on detection probability of male bellows in nightly recordings; and (iii) determined the effective sampling area of a recording unit.

## Materials and methods

**Fig 1:**
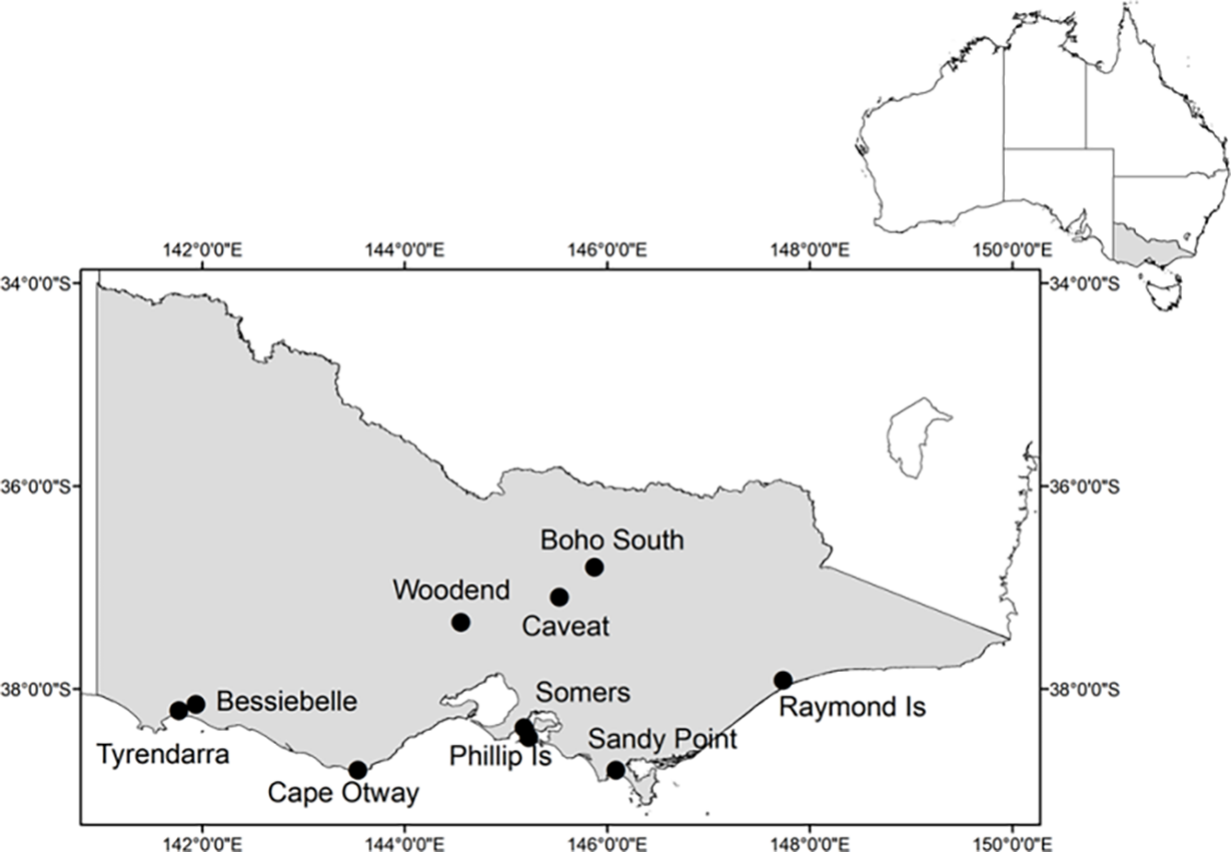
The locations of the study sites in Victoria, Australia where autonomous recording units were deployed from August 2016 to January 2017.

Ten study sites were chosen within the koala’s southern range in Victoria, Australia (Fig 1).

### Koala population density

Koala population density at each site (except Phillip Island) was assessed using the Double Count Transect method [29], undertaken on one day during December 2016 or January 2017. Because koala population densities do not show much monthly variation, it is standard protocol to conduct annual monitoring during the breeding period [17]. Either one 500m or two 250m transects separated by 50m were used depending on the habitat configuration. Two observers independently searched for koalas within 25m of transects and their counts were used to determine the density (koalas/hectare) in the site. At Phillip Island, the site was within the Koala Conservation Centre where the number of koalas is known.

### Autonomous recording units

One autonomous recording unit (Song Meter: SM3 or SM4, Wildlife Acoustics, Maynard, Massachusetts, USA) was deployed at each site from August 2016 to January 2017 inclusive to encompass the breeding season of southern koalas [30, 31]. Each Song Meter was mounted 1.8m above ground on a tree within the site. The units were powered with internal batteries (1.5 V, D-Cell, alkaline) that were replaced every six weeks, and recordings were stored on Secure Digital (SD) cards (32 GB or 64 GB) in uncompressed wave format. Each unit used two built-in omnidirectional, stereo microphones with a dynamic range of 14 dB to 94 dB SPL at 0Db gain.

We programmed each recording unit to record for five minutes at the beginning of each hour between 02:00h and 19:00h, and continuously for six hours from 20:00h to 02:00h for each day of the deployment. This schedule allowed us to document the diel cycle, and the weekly variation in night-time vocalisations during the breeding season. Our decision to record continuously for six hours at night was based on an accelerometry study of free-ranging koalas during the breeding season [32]. In that study, koalas showed a distinct diel pattern of activity, with most activity occurring from mid-afternoon to early morning, and peaking between 20:00h and 02:00h. Although bellowing behaviour was not specifically recorded, we assumed that it would be correlated with timing of activity. The audio sample rate was set at 24,000 Hz, and no bias or filters were enabled for recordings.

### Audio data processing

**Fig 2:**
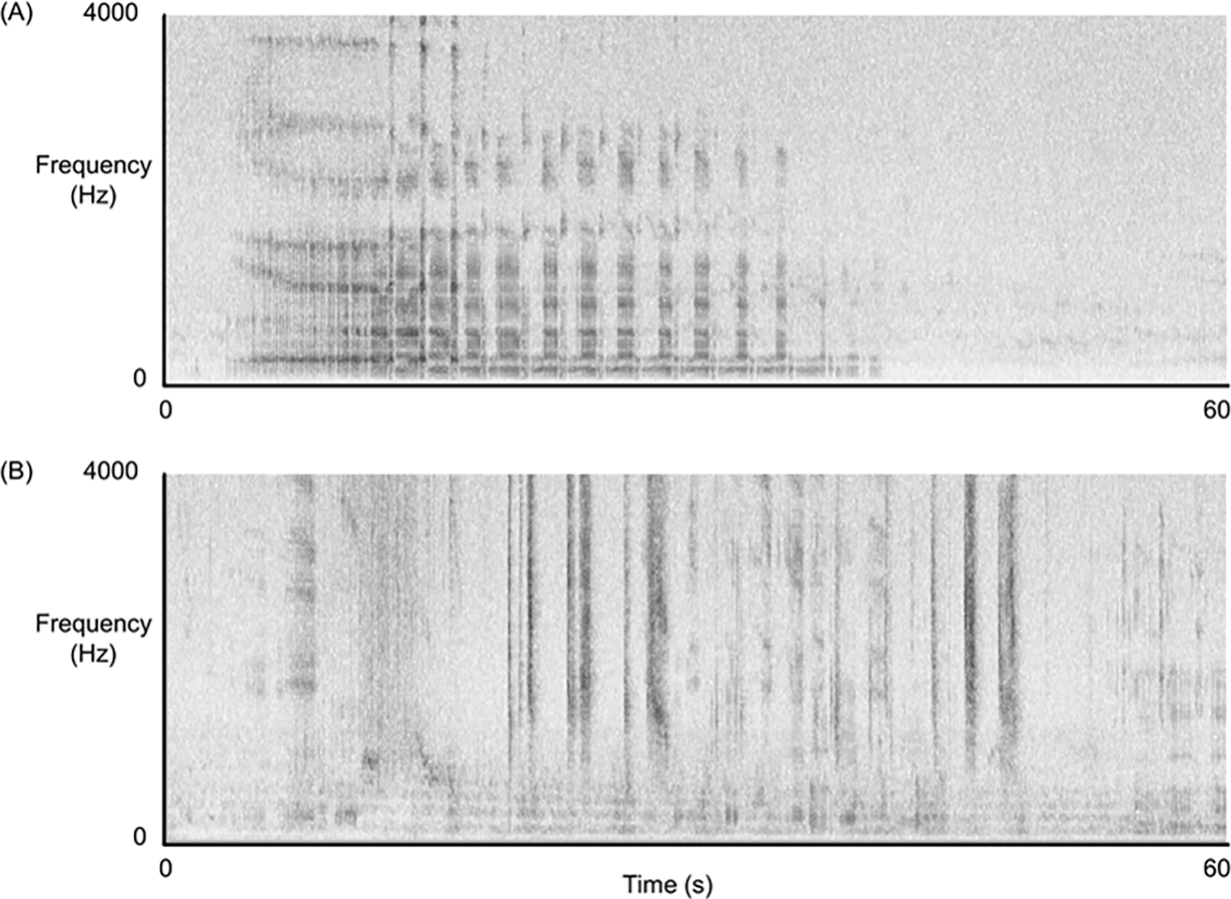
Spectrograms of (A) Male bellow characterised by an introductory phase followed by short inhalations and exhalations; and (B) Female vocalisation.

We manually inspected spectrograms of recordings for koala vocalisations (Fig 2; Audacity, Version 2.0.2, 1999–2015, http://audacity.sourceforge.net/). We recorded the time of onset of each male vocalisation (‘bellow’) in a recording. Bellows usually were discrete with the onset or introductory phase clearly defined (Fig 2A). When male bellows overlapped, we listened to recordings to determine the number of males bellowing. Female vocalisations were easily distinguished from those of males. They were not as discrete as males, often continuing throughout an entire one hour recording. We therefore only recorded their presence in a recording. We determined the most effective spectrogram preferences for visually detecting vocalisations in Audacity (Table 1) from a subset of recordings, and used a 5-minute viewing frame. Recordings were processed by seven trained observers. The ability of each observer to detect koala vocalisations was tested using six randomly-selected one-hour recordings per observer. We did not process recordings that were taken during inclement weather due to our inability to detect vocalisations in those spectrograms. This resulted in no data for a maximum of three consecutive days and a total of nine days per site.

**Table 1:** Spectrogram preferences in Audacity 2.0.2 used to visually detect koala vocalisations.

Parameter	Setting
Scale	Linear
Minimum Frequency (Hz)	0
Maximum Frequency (Hz)	4000
Gain (dB)	20
Range (dB)	80
Frequency gain (dB/dec)	0
Algorithm	Frequencies
Window Size	4096

### Detection distance for vocalisations

To determine the potential detection area of a recording unit in our study, we conducted a playback study in two habitat types: (i) open forest with shrub understorey; and (ii) open grassland. The open forest site was chosen as similar to many of the forested sites used in the study. The test was repeated in the open area to determine the influence of vegetation on detection distance. Testing was undertaken under fine weather conditions and wind speed of 0 between 11:00h and 14:00h. Recordings of a male bellow and female vocalisation obtained from one of the sites were played from a Chaiyo Focus 505 loudspeaker (Taipei, Taiwan) at 1.5m above ground, and at a sound pressure level of a naturally bellowing male (75 dB at 1m from the speaker [27]). We recorded the playbacks with a Song Meter (SM4) held at 1.8m above ground at a distance of 1m and then at 20-m intervals from the loudspeaker, to a distance of 140m. The Song Meter was programmed to record continuously at the same sampling rate (24,000 Hz) of Song Meters deployed at sites. At each distance, we measured sound levels with a Jaycar Compact Digital Sound Level Meter, set for C-weighted fast response (Jaycar Electronics, Australia). We viewed spectrograms of the recordings to determine the distance at which we could no longer visibly detect vocalisations.

### Data analysis

#### Diel and weekly variation in bellow occurrence

To examine the diel cycle in bellow occurrence we used the number of bellows in each of the hourly 5-minute recordings and from the first five minutes of each hour between 20:00h and 02:00h. Because koala activity is linked with time of sunset [32] and the time of sunset varied from 17:36h in August to 19:54h in January, for each day we converted time to ‘hours from sunset’. Hours from sunset were positive for the 11 hours after sunset, and negative for the 12 hours before. For each site, we then used the total number of bellows per ‘hour from sunset’. Because of the variation between sites in the number of bellows recorded, we standardised this number to the proportion of the total number of bellows recorded per 24 h per site.

To examine weekly variation in bellow occurrence, we used the number of bellows in each nightly six-hour continuous recording to calculate the mean number of bellows per night hour per week per site. We considered that the six-hour recordings would provide a more accurate assessment of bellow occurrence than the five-minute recordings, especially at low population density sites. To standardise across sites, we expressed this number as the proportion of the total mean number of bellows per night hour per week per site.

Generalised Additive Models (GAMs) were used to investigate the relationship between bellow occurrence and hour from sunset, and bellow occurrence and survey period (week). GAMs were run with an underlying Gaussian distribution and residual plots were used to assess model fit and appropriateness of the models’ distribution. Diel bellow occurrence was modelled using ‘hour from sunset’ with a smoothing parameter allowing for a non-linear distribution. A fixed variance structure was added to account for increasing variability in bellow occurrence as ‘hour from sunset’ increased. Weekly bellow occurrence was modelled using ‘survey week’ with a smoothing parameter allowing for a non-linear distribution. A fixed variance structure was added to ‘survey week’ to account for the heterogeneous pattern in the residuals. Three sites (Sandy Point, Somers and Woodend) were excluded from this analysis due to the low number of bellows detected (less than 225 bellows per site for the entire study period).

#### Detection probability

Single-species single-season occupancy models were used to determine the influence of stage of the breeding season, koala population density, and sampling schedule on koala detection probability [33]. Models were run for three stages of the breeding season (early: August/September; mid: October/November; late: December/January); and for low (<1 koala/ha) and high (≥1 koala/ha) population densities. We used data from the 6-h recording period for this analysis to determine the influence of sub-sampling during this period when bellowing activity peaks. Sub-sampling schedules tested were: (i) 30 minutes per hour, (ii) three hours around the time of peak bellowing, (iii) two hours around the time of peak bellowing, and (iv) one hour at the time of peak bellowing. Separate models were used because there were only ten sites and the inclusion of covariates over-parameterised the models. Within each model, both site occupancy and detection probability were held constant. The number of survey nights required to be 80 and 95% confident in a site-specific absence were calculated using the formula:

$$P=1‑\left(1‑p_{1}\right)\;*\;\left(1‑p_{2}\right)\;*\;\left(1‑p_{3}\right)\;\ldots \;\left(1‑p_{n}\right)$$

Where *P* is the cumulative nightly detection probability, *p*_1_ is the detection probability for night one, and *n* is the total number of survey nights. All sites were used in this analysis.

All analyses were conducted in R [34] and using the package ‘mgcv’ for GAMs [35] and ‘unmarked’ for occupancy modelling [36].

## Results

A total of 21,241 male bellows were detected in 9,007 hours of recordings. Female screams were detected in 2.9% of recordings, and at all sites except Woodend. Because of their low occurrence, female ‘screams’ were not considered further. Male bellow occurrence varied between sites and was positively correlated with the double-count estimate of koala population density (*Pearson’s r* = 0.831, *P* = 0.003; Table 2). The number of recording days was influenced by inclement weather, and intermittent failure of recording units (presumably due to batteries). The latter primarily affected the number of recording days at Woodend and Sandy Point.

**Table 2:** Summary of koala population density (from double count transect survey), the occurrence of male bellows, and presence of female vocalisations in recordings from autonomous recording units deployed at ten study sites in Victoria between August 2016 and January 2017. Double-count estimates of population density were made at all sites except Phillip Island for which koala density data was provided by the Koala Conservation Centre.

Site	Population density (koalas/ha ± SE)	Number of recording days	Number of days with male koala vocalisations	Bellow occurrence (bellows/h; mean ± SE)	Days with female screams present (%)
*Low density (<1 koala/ha)*					
*Tyrendarra*	0.25 ± 0	130	103	1.50 ± 0.07	11.6
*Woodend*	0.30 + 0	114	7	0.03 ± 0.02	0.0
*Caveat*	0	133	122	2.17 ± 0.09	18.3
*Boho South*	0.97 ± 0.80	134	104	1.51 ± 0.08	11.3
*Somers*	0.3 + 0	144	33	0.15 ± 0.04	2.7
*Sandy Point*	0.69 + 0	103	49	0.33 ± 0.03	3.8
*High density (≥1 koala/ha)*					
*Raymond Island*	1.00 + 0	119	116	2.55 ± 0.11	27.7
*Bessiebelle*	1.13 + 0.49	124	120	5.33 ± 0.21	30.7
*Cape Otway*	7.19 ± 1.07	134	129	8.89 ± 0.34	61.9
*Phillip Island*	3.00	131	128	3.80 ± 0.12	65.2

### Diel pattern in bellow occurrence

**Fig 3:**
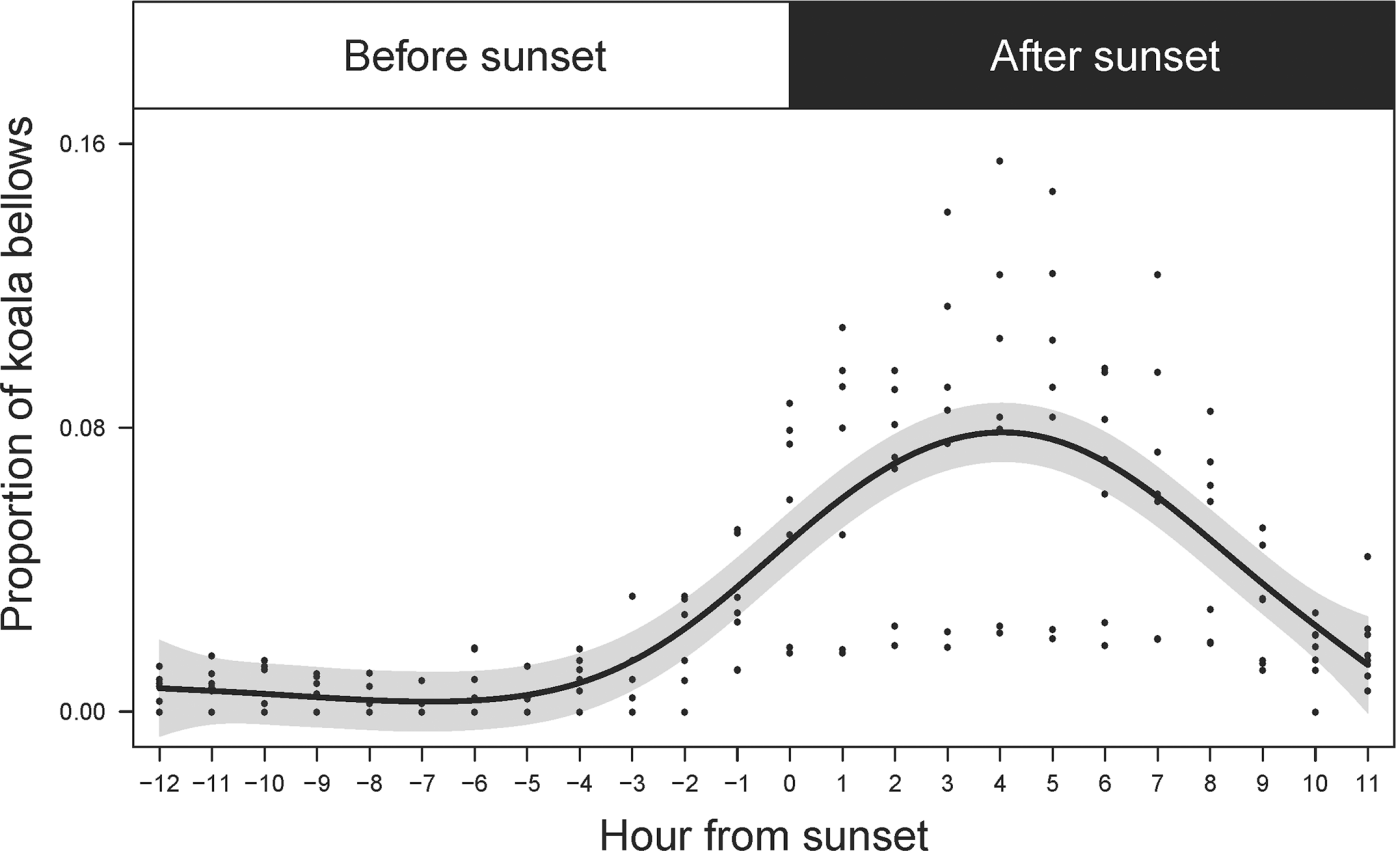
Diel cycle of male koala bellows from recording units deployed at seven sites in Victoria. Recording units recorded for five minutes/hour at each site and were deployed from August 2016 to January 2017. The solid line is the predicted proportion of bellows per 24-hour period and shaded area represents the 95% confidence intervals of the mean.

Male bellow occurrence was lowest during daylight hours, increasing three hours prior to sunset and peaking at four hours after sunset (Fig 3). The additive relationship had strong explanatory power explaining 65% (R^2^ = 0.649) of the variability in the data.

### Weekly pattern in bellow occurrence

**Fig 4:**
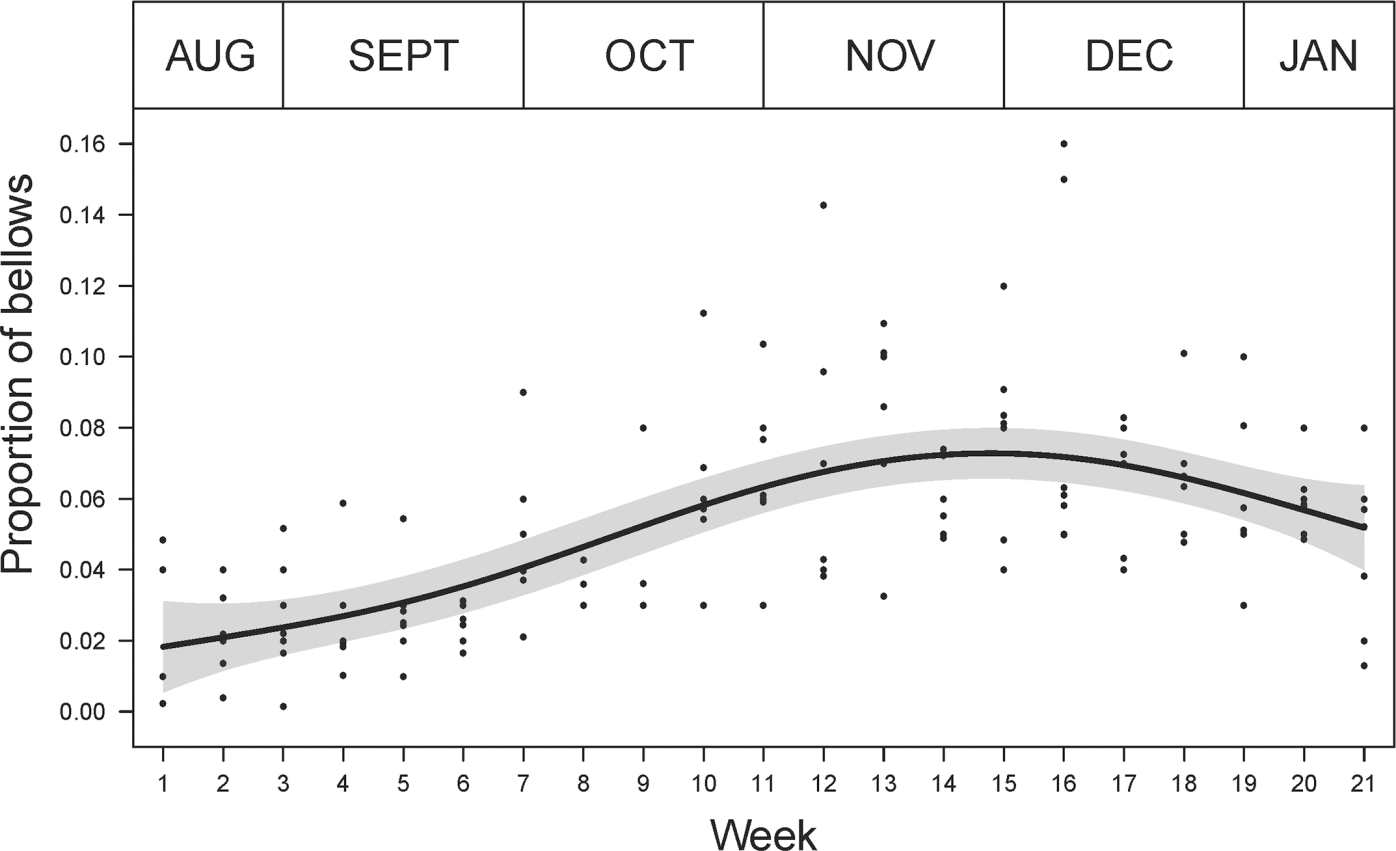
The pattern in male koala bellow occurrence during the breeding season (August to January). The solid line represents the predicted proportion of total bellows recorded during this period and shaded area shows the 95% confidence interval of the prediction.

Male bellow occurrence was relatively low during August and September, increasing in October and peaking in late November (Fig 4). The additive relationship had moderate explanatory power explaining 43% (R^2^ = 0.434) of the variability within these data.

### Detection probability

Nightly detection probability (recording between 20:00 and 02:00h) was high at high koala density sites, with between one and three nights of monitoring required to be 95% confident of a site-specific absence, depending on the season and sampling schedule (Table 3).

**Table 3:** Estimated nightly detection probabilities (*P*) with standard error (*SE*) for male koala bellows relative to stage of the breeding season (early, mid- and late), sampling schedule, and koala population density (low: <1 koala/ha, and high: ≥1 koala/ha). The number of nights required to be 80% and 95% confident of a site-specific absence of koalas is given.

		Low density (<1 koala/hectare)				High density (≥ 1 koala/hectare)			
Stage of breeding season	Nightly sampling schedule	*P*	*SE*	80%	95%	*P*	*SE*	80%	95%
Early	6 hours	0.385	0.031	4	7	0.962	0.015	1	1
	6 x 30 minutes	0.333	0.030	4	8	0.924	0.021	1	2
	3 hours	0.317	0.029	5	8	0.874	0.026	1	2
	2 hours	0.274	0.028	6	10	0.778	0.033	2	2
	1 hour	0.280	0.031	5	10	0.646	0.038	2	3
Mid	6 hours	0.558	0.030	2	4	0.947	0.017	1	2
	6 x 30 minutes	0.504	0.030	3	5	0.941	0.018	1	2
	3 hours	0.561	0.033	2	4	0.924	0.020	1	2
	2 hours	0.465	0.033	3	5	0.894	0.024	1	2
	1 hour	0.368	0.032	4	7	0.835	0.028	1	2
Late	6 hours	0.793	0.030	2	2	0.988	0.009	1	1
	6 x 30 minutes	0.690	0.034	2	3	0.969	0.014	1	1
	3 hours	0.685	0.034	2	3	0.951	0.017	1	1
	2 hours	0.582	0.036	2	4	0.890	0.025	1	2
	1 hour	0.457	0.037	3	5	0.834	0.029	1	2

**Fig 5:**
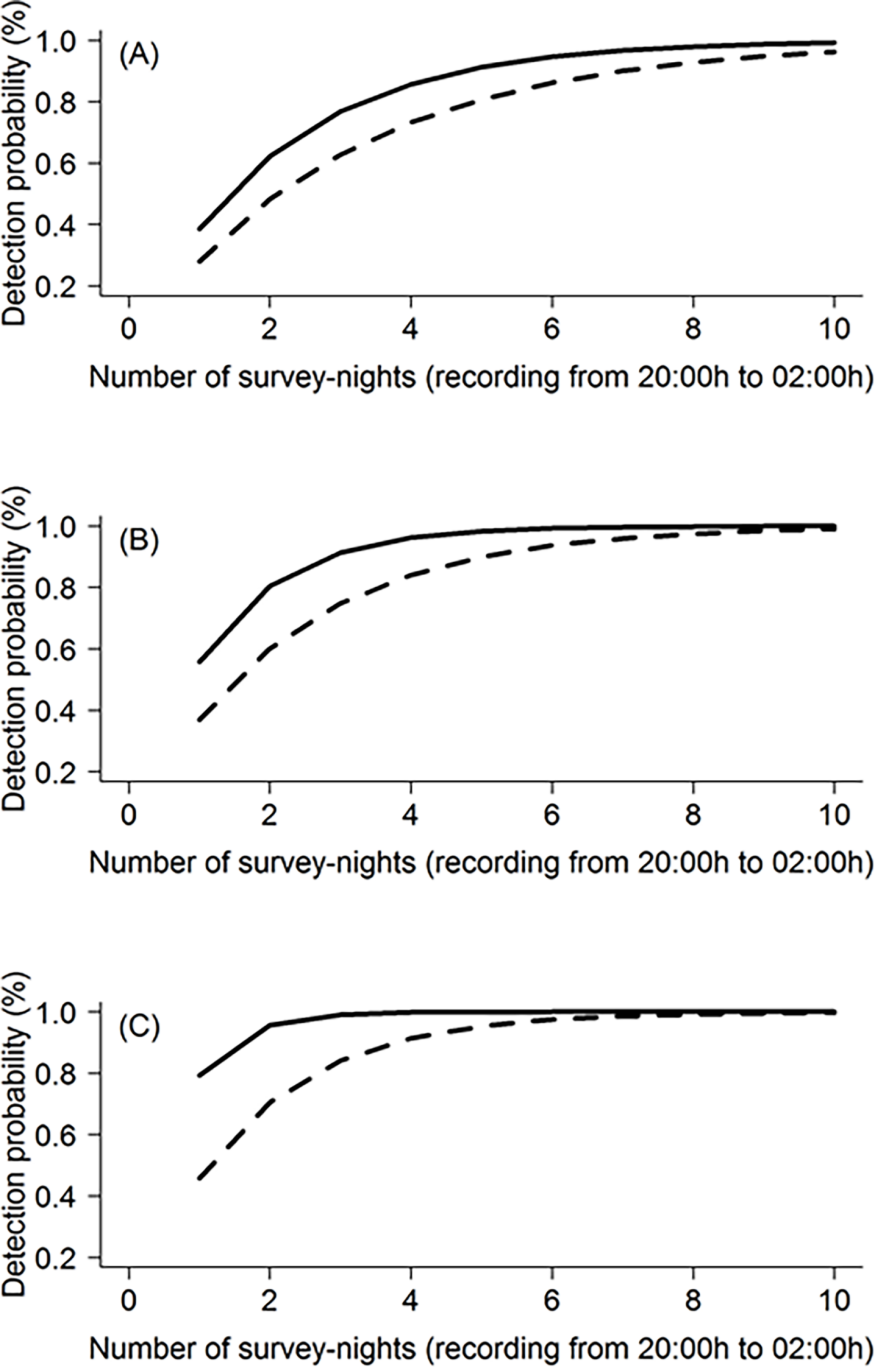
Detection probability versus the number of survey nights required to be confident in site-specific absences of male koala bellows. Detection probabilities were considered for low koala population density (<1 koala/ha) during the (A) early breeding season (August/September), (B) mid breeding season (October/November), and (C) late breeding season (December/January). The dashed line represents the sampling schedule with lowest nightly detection probability (one hour per night), and the solid line represents that associated with the highest nightly detection probability (six hours per night).

At low population densities (<1 koala per hectare), between 2 and 10 nights of monitoring was required to be 95% confident of a site-specific absence of koalas, depending on the stage of the breeding season and sampling schedule (Table 3; Fig 5). Stage of the breeding season rather than sampling schedule had the strongest influence on detection probability (ANOVA; Season: F_2,12_ = 16.02, *P* = 0.0004, AIC = -24.859; Sampling schedule F_2,12_ = 0.698, *P* = 0.61, AIC = -5.054), with the most nights required during the early breeding season (7–10 nights) and least nights required during the late breeding season (2–5 nights) to be 95% confident of a site-specific absence of koalas. Although six hours of continuous recording always was associated with the highest detection probability and lowest number of sampling nights required for detection confidence, high confidence could still be achieved by reducing the nightly sampling effort and increasing the number of sample nights (to a maximum of ten nights during the early breeding season; Table 3).

### Detection distance of recorders

Sound level (dB) declined with increasing distance from the playback speaker and became lower than the ambient noise level (42 dB) at 100m. Sound levels of male bellows or female vocalisations relative to distance from the speaker did not vary between the open forest and open grassland site (*t* = 1.154, *df* = 6, *P* = 0.292). Habitat type influenced the visibility of bellows and vocalisations in spectrograms. In open forest, spectral images of male bellows began to degrade at 100m, compared to 120m in open grassland.

## Discussion

Our study highlights the importance of understanding the factors influencing the pattern of vocalisations of a species when designing acoustic monitoring programs. Without such an understanding, monitoring programs may fail to detect species and thereby result in inaccurate predictions of species’ distributions. For koalas in the southern part of their range, we identified clear diel and weekly patterns in male koala bellows during the breeding season. Female ‘screams’ occurred only in a small proportion of recordings and therefore were not considered further. Occupancy modelling indicated that the stage of the breeding season (early, mid and late) had a greater influence on male detection probability than the nightly sampling schedule. At sites with low koala population density (<1 koala per hectare), between seven and ten nights (six hours of recording per night) during the early breeding period was required to be 95% confident in a site-specific absence whereas only two to five nights was required in the late breeding season. Our playback tests suggest that the detection area of a Song Meter is approximately three hectares (male bellows are easily seen in spectrograms at distances of 100 m). We were unable to test the influence of covariates (e.g. weather, topography etc) on bellow detection probability due to the small number of sites (*N* = 10) monitored. However, our results provide important information to guide the design of bioacoustics surveys for koalas, and we recommend a similar approach for developing guidelines for passive acoustic survey of other terrestrial species.

In our study, male koala bellow occurrence varied both with week during the breeding season and time of day. The occurrence of bellows increased following the first week of recorder deployment in August, reached a peak in November, and declined until recorder removal in January. Male bellows contain information about the identity of the male [27] and advertise the male’s location to other koalas in their range, serving both to avoid male-male interactions [22] and to attract females [23]. Consequently, an increase in bellow occurrence is considered to reflect an increase in breeding activity in a population [23, 25, 37]. The presence of female ‘screams’ in recordings confirms that males were interacting with females. Our study was timed to coincide with the breeding season of southern koala populations, which generally extends from September to January with some variation between sites [30] and years [31]. Studies on birth dates for koalas in their southern range have suggested that for most years a large proportion of births occur between November and January [30, 31]. Given the average gestation period for a female koala is approximately 35 days [38], the timing of peak bellowing activity we observed coincides with when most copulations are likely. The pattern we observed across multiple sites also suggests that the timing of the breeding period is relatively synchronous across the southern range of the koala.

Male koalas exhibited a distinct diel pattern of bellowing activity. Bellow occurrence was low from early morning (approximately 10 hours after sunset) to early afternoon (three hours before sunset), after which it increased to a peak at four hours after sunset. This pattern was expected based on koala activity patterns [32], and was the basis for choosing to record continuously for six hours at night to examine the seasonal variation in bellowing activity. Koalas primarily are nocturnal and numerous factors including the change in light intensity, temperature, and humidity, during the early evening hours likely explain the increase in activity at this time [32]. The energetic cost of male koala bellows is unknown; however, in other species there is an association between individual metabolic rate and vocalisations [39], with some studies suggesting it is an energetically expensive activity [23, 40]. If the energetic cost of bellowing is high, it would be most efficient for a koala to bellow during the night when temperatures are cooler, as this would help offset thermoregulatory stress that is associated with hot weather. Indeed, this trade-off would be beneficial considering that peak bellowing appears to occur in the warmest months of the year (November to December). The primarily nocturnal pattern of bellowing in our study has been observed elsewhere, although with some local variation in peak activity. In a study of koalas on French Island, Victoria, two peaks were observed: one just after sunset and another after midnight [37]. In north Queensland, peak bellowing was observed at midnight [23].

As expected, the probability of detecting koalas at a site was affected by koala population density, with detection probability high at high density (≥1 koalas/ha) compared to low density (<1 koala/ha) sites, irrespective of other factors. Even with a small sampling effort (e.g., one hour recording per night) during the early breeding season when bellows are least frequent, just three nights of sampling is required for detection confidence. However, at low population densities (<1 koala per hectare) which are more typical for much of the koala’s range, up to ten nights of sampling is required depending both on stage of the breeding season and sampling schedule. offstage of the breeding season had a greater influence on detection probability than sampling schedule with the late breeding season (December/January) being the most effective time to sample, and the early breeding season (August/September) being the least effective. Even with high sampling effort (recording for six hours per night), ten nights of sampling are required in the early breeding season as opposed to only two nights during the late breeding season or four nights during the mid-breeding season to be 95% confident in a site-specific absence of koalas. A different pattern of bellowing activity was observed in New South Wales [18] with detection probability declining slightly from October through to December. This may reflect some geographic variation in breeding and therefore bellowing activity, across the koala’s range. The potential for such variation therefore should be considered in the design of PAM for koalas across their range.

A sampling schedule that involves recording for six continuous hours from 20:00 to 02:00h is supported by the diel pattern in bellows we observed across sites. Analysis of detection probabilities indicates that subsampling during that period may be more efficient than continuous recording. Even at low population densities, sampling for one hour per night for five nights (5h total recording time) has equivalent detection probability to sampling for six hours per night for two nights (12h total recording time). Subsampling has obvious benefits, reducing power use and data storage requirements, as well as the processing time needed for recordings.

From these observations, passive acoustic monitoring may be efficient for tracking temporal changes in koala population distribution, requiring little sampling effort for high detection confidence irrespective of population density. Furthermore, our distance detection tests suggest that only one autonomous recorder may be required to monitor a three-hectare area. However, this assumes ideal weather conditions (wind speed of 0 and no rain), that the habitat is relatively uniform and open, and does not take into account the height above ground of a bellowing koala. In a test of sound transmission in different landscapes and vegetation types, one study found that sound detection spaces were highly variable [15]. In addition to local site factors, the height of the sound source had a non-linear effect on detection distance. We therefore recommend that further testing be undertaken to understand the influence of weather and site characteristics on detection distance of koala bellows. In sites where detection distance is relatively low, it may be necessary to deploy arrays of autonomous recorders rather than a single recorder.

Based on our results, we recommend the following design (timing and duration) of passive acoustic surveys for koalas:

- Surveys should be undertaken during the stage of the breeding season when koala bellowing activity is consistently high. In our study in the southern range of the koala, this is December/January. Although the timing of the breeding season is relatively synchronous throughout the range of the koala [41] and between years [31], bellowing activity may vary between regions [18]. If the timing of breeding and bellowing activity at a site is uncertain, the number of recording days should be increased to ensure confidence in detection.
- Recording units should be programmed to record for one hour (beginning four hours after sunset) and deployed for seven days during the mid- or late breeding season. During the mid-breeding season, this sampling strategy has the same probability of detection as recording for six continuous hours for four days, yet has 30% of the cost in terms of total recording time.
- If poor weather conditions that impacts on the quality of recordings is likely, the duration of recorder deployment should be extended.
- A detection distance test should be undertaken for each site type. This will provide a basis for determining the number of recorders to be deployed at a site, and for comparing results across site types.

If recording units are deployed according to these recommendations, results of surveys using passive acoustic recordings may be associated with fewer false-negatives than traditional methods of koala survey. Furthermore, because bioacoustic surveys are both real-time and a permanent record of sounds at a site, survey results can be checked retrospectively. This may be important where automated detection is used to analyse recordings. Although we manually scanned spectrograms for bellows to ensure high rates of detection, automated detection of male bellows in recordings has the potential to further improve the efficiency of bioacoustics in monitoring programs. In studies of northern koalas, [18, 23] demonstrated that automated detection is possible; however, the recogniser used in their study is not effective for detecting bellows of southern koalas (Whisson, D. A. 2016, Personal communication). Geographic variation in the acoustic characteristics of a terrestrial species has not been reported but warrants further attention in the development of recognisers for automated detection of species with broad geographic ranges.

In addition to using passive acoustic monitoring for determining presence/absence of male koalas at a site, this method also may have application for determining population density in a site and warrants further research. At a basic level, the positive correlation we observed between the number of bellows per hour and the double-count estimate of population density at a site suggests that PAM may provide a relative index of abundance. However, because PAM is only effective in detecting males, its application to estimating abundance assumes an equal sex ratio which may not be true of many sites. More sophisticated approaches also may be feasible. Methods have been developed to estimate density from acoustic data [2], and generally are based on mark-recapture theory (relies on identifying individuals), or spatially-explicit models that combine recapture and distance sampling models. Because male koala bellows contain information about the size of the koala, it is possible to identify individuals in recordings and therefore determine the number of male individuals present at a site. However, this would require deployment of multiple autonomous recorders at a site due to the degradation of identity information over short distances (approx. 50m [42]).

## Conclusions

In this study, we demonstrate that when designing an acoustic survey for a terrestrial species, it is critical to first have an understanding of the factors influencing the species’ seasonal and diel vocalisations. When this information is unavailable or there is a high degree of uncertainty, pilot studies should be undertaken and occupancy modelling used to determine the most effective design. Using this approach, researchers and managers can optimise resources while also having a high degree of confidence that their design is effective.

## Supporting information

S1 File Processed data from all recordings including site, time, and vocalisations present. Data is presented in worksheet ‘Data’ and metadata in worksheet ‘Codes’.
(XLSX)
